# Neuroprotective effects of FK866 against traumatic brain injury: Involvement of p38/ERK pathway

**DOI:** 10.1002/acn3.51044

**Published:** 2020-04-17

**Authors:** Zhongju Tan, Lili Chen, Yucheng Ren, Xiaohang Jiang, Wei Gao

**Affiliations:** ^1^ Department of Geriatrics The First Affiliated Hospital of Zhejiang University School of Medicine Hangzhou Zhejiang China; ^2^ Department of Neurology Xiasha Campus Sir Run Run Shaw Hospital Zhejiang University School of Medicine Hangzhou Zhejiang China; ^3^ Department of Neurosurgery The Second Affiliated Hospital of Zhejiang University School of Medicine Hangzhou Zhejiang China; ^4^ Department of Neurology Changxing People’s Hospital The Second Affiliated Hospital of Zhejiang University Changxing Campus Changxing Zhejiang China

## Abstract

**Objective:**

FK866 is an inhibitor of nicotinamide phosphoribosyltransferase (NAMPT), which exhibits neuroprotective effects in ischemic brain injury. However, in traumatic brain injury (TBI), the role and mechanism of FK866 remain unclear. The present research was aimed to investigate whether FK866 could attenuate TBI and clarified the underlying mechanisms.

**Methods:**

A controlled cortical impact model was established, and FK866 at a dose of 5 mg/kg was administered intraperitoneally at 1 h and 6 h, then twice per day post‐TBI until sacrifice. Brain water content, Evans blue dye extravasation, modified neurological severity scores (mNSS), Morris water maze test, enzyme‐linked immunosorbent assay (ELISA), immunofluorescence staining, and western blot were performed.

**Results:**

The results demonstrated that FK866 significantly mitigated the brain edema, blood–brain barrier (BBB) disruption, and ameliorated the neurological function post‐TBI. Moreover, FK866 decreased the number of Iba‐1‐positive cells, GFAP‐positive astrocytes, and AQP4‐positive cells. FK866 reduced the protein levels of proinflammatory cytokines and inhibited NF‐κB from translocation to the nucleus. FK866 upregulated the expression of Bcl‐2, diminished the expression of Bax and caspase 3, and the number of apoptotic cells. Moreover, p38 MAPK and ERK activation were significantly inhibited by FK866.

**Interpretation:**

FK866 attenuated TBI‐induced neuroinflammation and apoptosis, at least in part, through p38/ERK MAPKs signaling pathway.

## Introduction

Traumatic brain injury (TBI) is defined as damage to the brain induced by external mechanical impact. The severity of TBI is predicated on consciousness alteration, organized as mild, moderate, or severe.^1^ It has been estimated that the annual number of patients suffering from TBI around the world has exceeded 50 million, which costs about 400 billion dollars each year.^2^ TBI is grouped into two stages: the primary injury which is caused by external impact, and secondary injury which is caused by a series of changes in neurochemistry, metabolism, cells, and molecules.^3^ The mechanisms involved in secondary injury include excitotoxicity, oxidative stress, neuroinflammation, mitochondrial dysfunction, axon degeneration, brain edema, blood–brain barrier damage, and cell death.^4^ Exploring novel agents that target secondary injury is still urgent for TBI clinical treatment.

Nicotinamide phosphoribosyltransferase (NAMPT) is a crucial enzyme in the cyclic biosynthetic pathway of nicotinamide adenine dinucleotide (NAD), serving as the catalyzer of the generation from nicotinamide (NM) to nicotinamide mononucleotide (NMN).^5^ Indeed, it has been postulated that NAMPT may participate in the regulation of inflammation and is responsible for many inflammatory diseases.^6^, ^7^ NAMPT was also involved in the regulation of apoptosis.^8^, ^9^, ^10^ FK866 is an inhibitor of NAMPT.^11^, ^12^, ^13^ Recently, it has been demonstrated that FK866 protects ischemic neuronal injury and spinal cord injury, which were achieved by inhibition of neuroinflammation and apoptosis.^14^, ^15^ However, whether NAMPT inhibitor FK866 attenuates neuroinflammation and apoptosis after TBI remains to be clarified.

This study aims to illuminate the role and possible underlying mechanisms of NAMPT inhibitor FK866 in neuroinflammation and apoptosis post‐TBI.

## Materials and Methods

### Animals

Adult male and female Sprague–Dawley rats weighing 300–330 g from SLAC Laboratory Animal Co., Ltd. (Shanghai, China) were adopted as experimental animals. Housing conditions of the rats were unified by temperature and humidity control and a 12 h light/dark shift. Consent to all animal experiments was given by the Institutional Animal Care and Use Committee of Zhejiang University and all the procedures complied with the Guide for *the Care and Use of Laboratory Animals* by National Institutes of Health.

### TBI model

The TBI model was established by controlled cortical impact (CCI) as described previously.^16^ The CCI model was shown in Figure S1. The rats were fixed on a stereotaxic apparatus. A midline incision was made to create a 5‐mm‐diameter craniotomy. The marked skull was cut and removed by a drill without damaging the dura mater. The CCI was accomplished vertically to the brain surface by a PinPoint™ Precision Cortical Impactor (Cary, NC, USA) according to the following parameters: 4‐mm‐diameter impact tip, impact velocity of 3 m/sec, an impact duration time of 120 ms, and 2 mm displacement of the brain. The rat was placed on a warming pad with a rectal thermometer to maintain its body temperature at 36.0–36.5°C. After the injury, the bone flap was immediately replaced, sealed, and closured the skin. Sham‐operated animals underwent the same procedures without performing CCI. The rats were put back into cages until complete recovery from anesthesia in a heated chamber. The rats in our study were carefully taken care, especially after surgery.

### Animals grouping and drug administration

One hundred and Fifty‐nine male rats and twenty‐four female rats were involved and assigned into three groups in random: sham group, TBI + vehicle group, and TBI+ FK866 group. The experimental outline was shown in Figure S2. The rats sacrificed in each group were shown (Fig. S3). FK866 (Sigma‐Aldrich, F8557) was dissolved in dimethyl sulfoxide (DMSO) and diluted in saline before use. FK866 (5 mg/kg) was administrated intraperitoneally at 1 h and 6 h after TBI. And the same dose of FK866 was given twice a day until sacrificed. The dose and the administration time of FK866 were selected based upon a previous publication.^14^

### Brain water content

The extent of brain edema was quantified by the wet weight/dry weight method. Twenty‐four hours post‐TBI, rats were euthanatized, and the brains were weighed for wet weight at once after removal. The dry weight of the brains was obtained after 24 hours of drying at 105°C. The brain water content was calculated as: [(wet weight − dry weight)/wet weight] × 100%.

### Evaluation of neurological deficits

The neurological deficits after TBI were assessed by the modified neurological severity scores (mNSS) including motor, sensory, reflex, and balance tests.^17^ The score was conducted and analyzed by an observer who was blinded to the experimental cohorts.

### Morris water maze test

Morris water maze test was conducted as previously described.^18^ Briefly, the rat was trained to find the platform before TBI or sham operation. For each trial, the rats were randomly placed into a quadrant start point facing the wall of the pool and were allowed a maximum of 60 sec to escape to the platform. Rats that failed to escape within 90 sec were placed on the platform for a maximum of 20 sec and returned to the cage for a new trial. Maze performance was recorded using a video camera suspended above the maze and interfaced with a video‐tracking system. The average escape latency of a total of five trials was calculated. The test was conducted at 3 and 7 days after TBI or sham operation. The test was conducted and analyzed by an observer who was blinded to the experimental cohorts.

### Evans blue dye extravasation

The Evans blue dye extravasation was conducted as previously described.^19^ Evans blue dye (2%, 5 ml/kg) was administrated via the left femoral vein and circulated for 1 hour. Under deep anesthesia, rats were sacrificed by cardiac perfusion. Then, removed and separated the brain to get the ipsilateral pericore parietal cortex immediately. Subsequently, weighted the brain samples and homogenized with 3 ml of 50% trichloroacetic acid, then centrifuged at 15,000*g* for 30 min. The supernatant was mixed with an equal volume of trichloroacetic acid with ethanol. After overnight incubation (4°C), the samples were centrifuged again (15,000 g, 30 min) and measured by spectrofluorophotometer (excitation wavelength 620 nm and emission wavelength 680 nm).

### Enzyme‐linked immunosorbent Assay

The levels of inflammatory cytokines were quantified using enzyme‐linked immunosorbent assay (ELISA) kits specifically for rats, following the instruction from manufacturers (Nanjing Jiancheng Bioengineering Institute, Nanjing, China). Briefly, the brain samples were homogenized in 1 ml of buffer containing 1mmol/L of phenylmethylsulfonyl fluoride, 1 mg/L of pepstatin A, 1 mg/L of aprotinin, and 1 mg/L of leupeptin in PBS solution with a glass homogenizer and then centrifuged at 12,000*g* for 20 min at 4°C. The supernatant was then collected and total protein was determined. The cytokine contents in the brain samples were expressed as a pictogram of antigen per milligram protein.

### Immunofluorescence staining and HE staining

After euthanasia at 24 h post‐TBI, the rats underwent intracardial perfusion with 0.1 mol/L PBS and 4% paraformaldehyde (pH 7.4). After, the brains were separated and preserved in 30% sucrose for 72 h. After sufficient dehydration, the brains were sliced coronally into frozen sections (7 μm) on a cryostat (Leica CM1950). HE staining was conducted following a standard protocol. For immunofluorescence staining, the primary antibody was monoclonal mouse anti‐NeuN (1:500, ab104224, Abcam), polyclonal goat anti‐Iba‐1 (1:200, ab506,Abcam)，monoclonal mouse anti‐GFAP (1:200, ab10062, Abcam), and monoclonal mouse anti‐AQP4 (1:100, ab9512Alexa Fluor 594 donkey anti‐mouse and donkey anti‐goat (1:500, Invitrogen) were used as the secondary antibody). Terminal deoxynucleotide transferase‐deoxyuridine triphosphate (dUTP) nick end labeling (TUNEL) was performed following the manufacturer’s protocol (Roche, Switzerland). Finally, the slices were covered by DAPI and observed under a fluorescence microscope. All procedures were conducted by two investigators blind to the experimental condition.

We selected at least three sections per rats with the ipsilateral pericore parietal cortex and three fields with a magnification of 200 × per section. For quantification of Iba‐1 and GFAP‐positive cells, the number from fields was averaged and expressed as positive cells per millimeter. For quantification of apoptotic neurons and AQP4‐positive cells, the percentage of TUNEL‐positive neurons or AQP4‐positive cells was calculated as follows: (the number of TUNEL‐positive neurons or AQP4‐positive cells/total number of neurons or cells) ×100%. Tissue sections were analyzed by an observer who was blinded to the experimental cohorts.

### Western blot

Western blot was conducted as described previously.^19^ Briefly, the ipsilateral pericore parietal cortex was collected. For protein extraction and isolation, a nuclear and cytoplasmic protein extraction kit (Beyotime, Jiangsu, China) was used following the protocol from manufacturer. Protein concentrations were determined by Detergent‐Compatible Protein Assay (Bio‐Rad). The same amount of each sample (60 μg) was separated by sodium dodecyl sulfate‐polyacrylamide gels and transferred to polyvinylidene fluoride membranes. Afterwards, the membranes were probed at 4°C overnight with antibodies against the proteins as follows: albumin (1:1000, Abcam, Ab106582), p‐38 (1:1000, Cell Signaling Technology, #8690), phospho‐p‐38 (1:1000, Cell Signaling Technology, #4511), phosphor‐ERK (1:1000, Cell Signaling Technology #9102), ERK (1:1000, Cell Signaling Technology #4370), NF‐κB p65 (1:5000, Abcam, Ab32536), Bcl‐2(1:500, Abcam, Ab59348), Bax (1:1000, Abcam, Ab32503), caspase 3 (cleaved form, 1:500, Abcam, Ab13847), monoclonal rabbit antioccludin (1:50000, ab167161, Abcam). For positive control of nuclear and cytoplasmic extracts, histone (H3) and β‐actin were selected, respectively. After primary antibody incubation, the membranes were further processed with corresponding horseradish peroxidase‐conjugated secondary antibodies at 21°C for 1 h. The protein bands were visualized using X‐ray film and analyzed by densitometry using ImageJ software (NIH).

### Statistical analysis

Band density values of the target proteins were normalized to the control group to enable comparisons between the different groups. Data were presented as mean ± SD or median (interquartile range) based on whether satisfying normal distribution or not. For data satisfying normal distribution, a significant difference between groups was analyzed with one‐way ANOVA followed by Tukey's multiple comparison tests. For data satisfying non‐normal distribution, comparisons between groups were performed by Mann–Whitney test. *P* < 0.05 was regarded as statistical significance.

## Results

### FK866 administration improved neurological function following TBI

**Figure 1:**
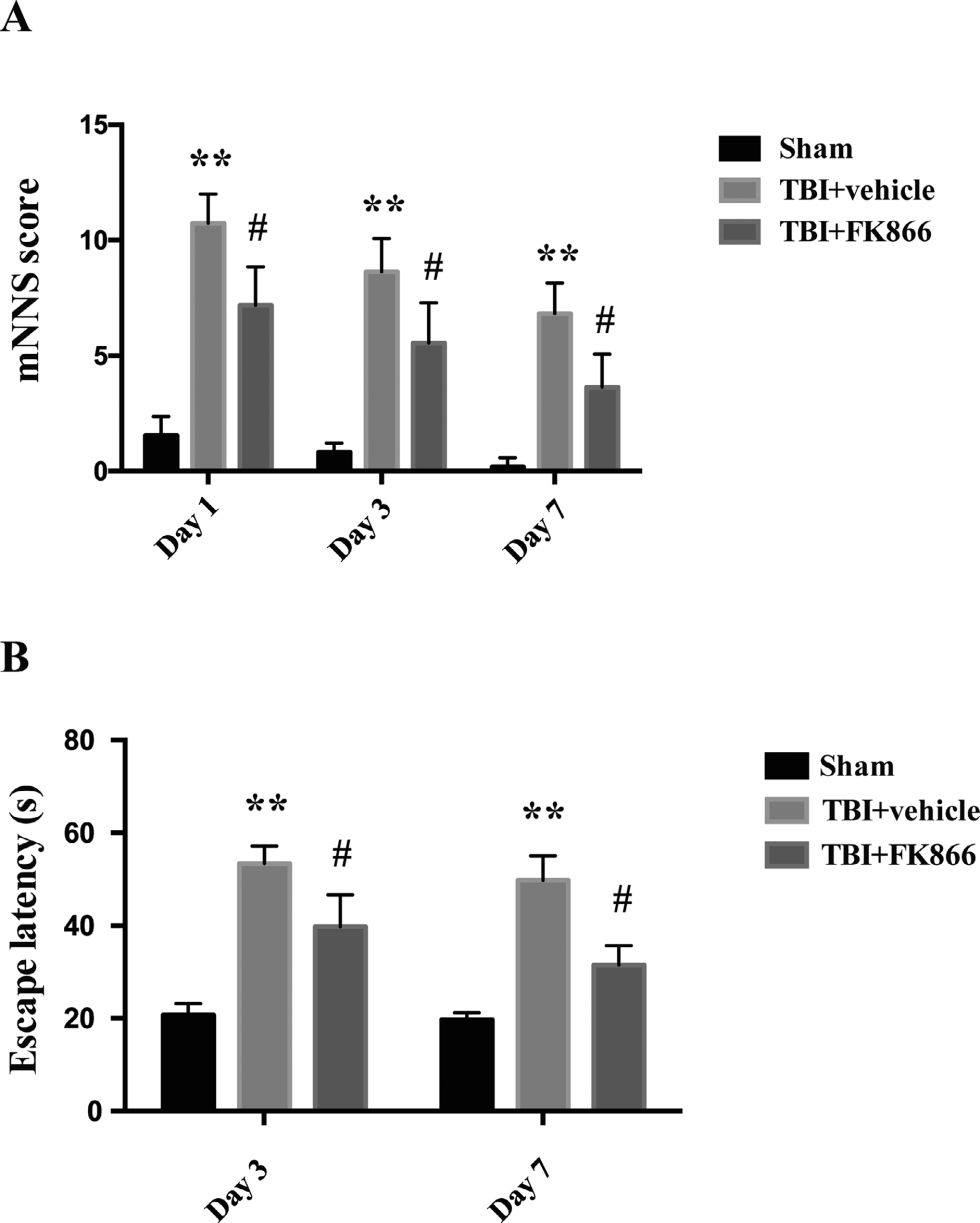
The effect of FK866 on neurological function after TBI. (A) The quantification of modified neurological severity score (mNSS) at 1, 3, and 7 days after TBI or sham operation. *n* = 59/group. The bar represents the mean ± interquartile range **P < 0.01 versus sham group; ^#^ *P* < 0.05 versus TBI + vehicle group. (B) The effect of FK866 on the escape latency performance via Morris water maze at 3 and 7 days after TBI or operation. *n* = 6/group/time point. The bar represents mean ± SD **P < 0.01 versus sham group; ^#^ *P* < 0.05 versus TBI + vehicle group.

The mNSS scores in TBI + vehicle group were significantly increased at day 1, day 3, and day 7 compared with that in sham operation (*P* < 0.01, Fig. 1A), FK866 administration treatment significantly decreased mNSS scores at day 1, day 3, and day 7 compared with the vehicle administration (*P* < 0.05, Fig. 1A). On day 3 and day 7, there was a significant increase in escape time among the TBI + vehicle group compared with sham controls (*P* < 0.01, Fig. 1B). However, the TBI‐induced deficits could be significantly attenuated by FK866 administration (*P* < 0.05, Fig. 1B). In addition, similar neuroprotective effects were also observed in the female rats after TBI (Fig. S4).

### FK866 alleviated brain edema and blood–brain barrier (BBB) following TBI

**Figure 2:**
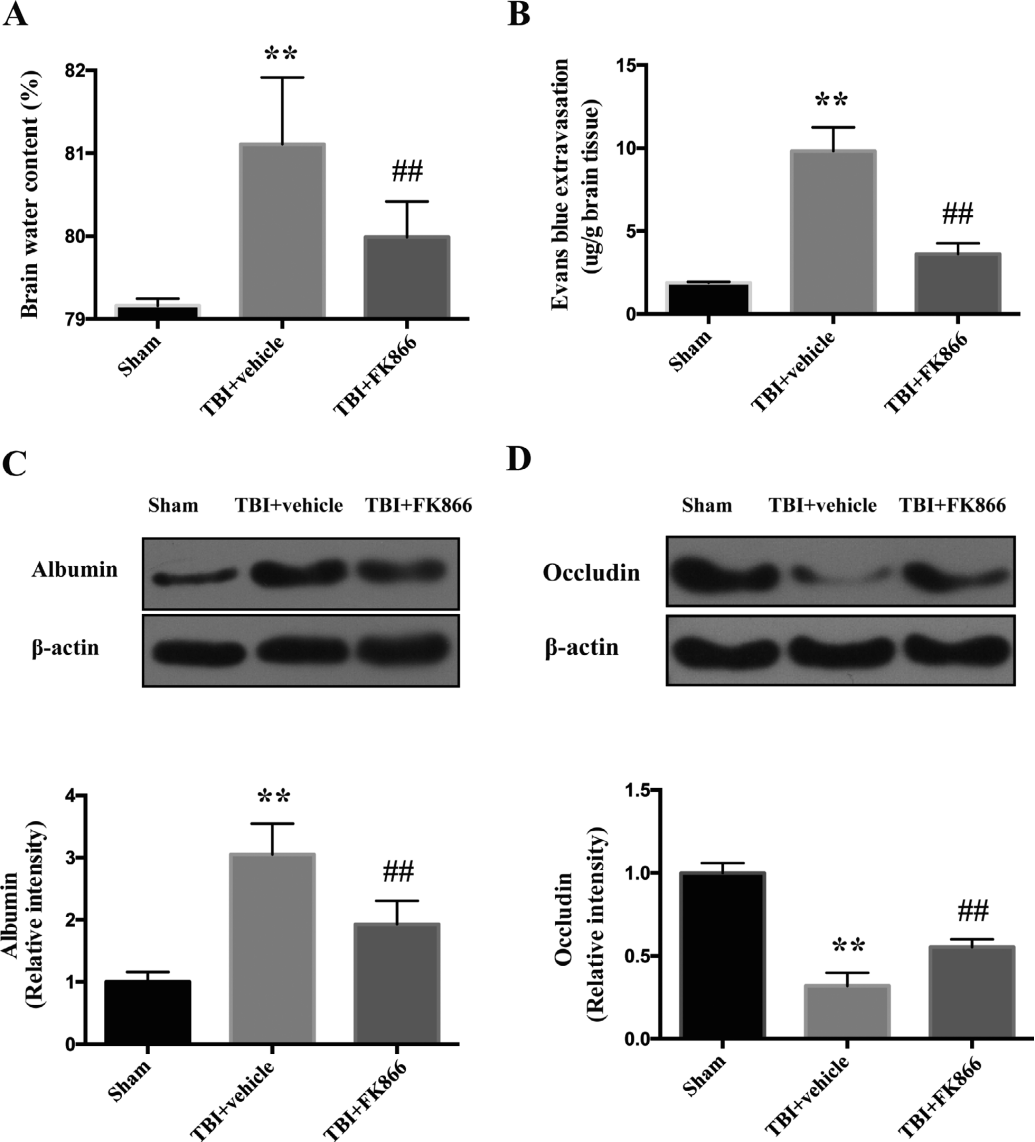
The effect of FK866 on brain edema, and BBB disruption at 1 day after TBI or sham operation. (A) Quantification of brain water content. The bar represents the mean ± SD. n = 6. The bar represents mean ± SD, **P < 0.01 versus sham, ^##^ *P* < 0.01 versus vehicle control. (B) Quantification of Evans blue dye extravasation. The bar represents the mean ± SD. *n* = 6. **P < 0.01 versus sham. ^##^ *P* < 0.01 versus vehicle control. (C) Representative western blots and Densitometric quantification of albumin. The bar represents the mean ± SD. *N* = 6. **P < 0.01 versus sham group; ^##^ *P* < 0.01 versus TBI + vehicle group. (D) Representative western blots and densitometric quantification of occluding. The bar represents the mean ± SD. *n* = 6. **P < 0.01 versus sham group; ^##^ *P* < 0.01 versus TBI + vehicle group.

Brain water content in the TBI + vehicle group was significantly increased compared with the sham control (*P* < 0.01, Fig. 2A), which was significantly reduced by FK866 administration (*P* < 0.01, Fig. 2A). Consistently, administration of FK866 led to a significant amelioration of Evans blue dye extravasation at 24 h post‐TBI (*P* < 0.01, Fig. 2B). Besides, the expression of albumin in the pericore cortex was significantly increased compared with the sham controls (*P* < 0.01, Fig. 2C), which was opposed to occluding protein expression (*P* < 0.01, Fig. 2D). FK866 administration significantly downregulated the albumin expression (*P* < 0.01, Fig. 2C) and upregulated the occluding expression compared with vehicle administration (*P* < 0.01, Fig. 2D).

### FK866 decreased the percentage of AQP4‐positive cells

**Figure 3:**
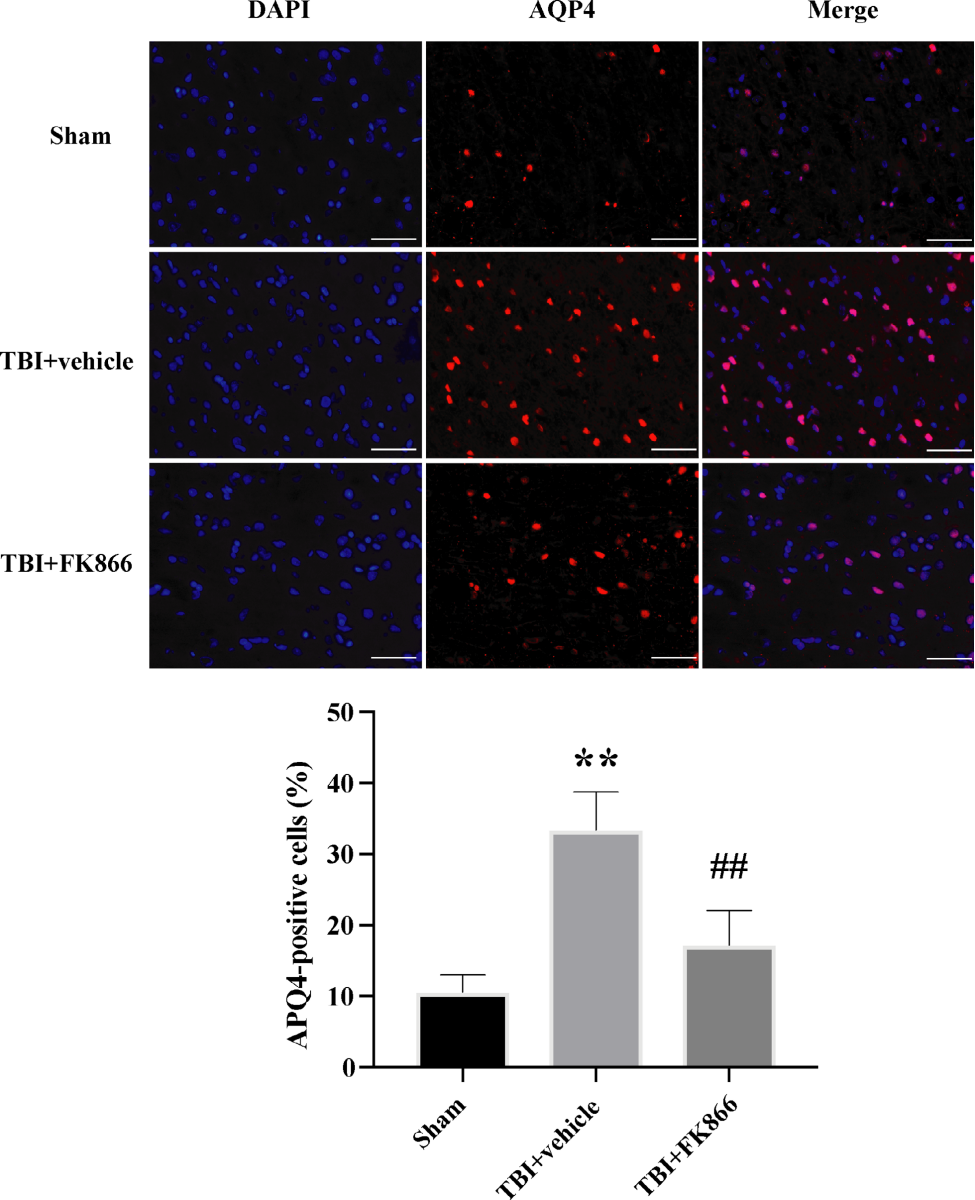
The effects of FK866 on the AQP4 expression at 1 day after TBI or sham operation. In the rats subjected to TBI, the percentage of AQP4‐positive cells was higher when compared with the sham group, while FK866 decreased AQP4 expression. Scar bar = 100 μm. *n* = 5/group. The bar represents mean ± SD, **P < 0.01 versus sham group; ^##^ *P* < 0.01 versus TBI + vehicle group.

The percentage of AQP4‐positive cells was significantly higher in the TBI + vehicle group than the control group (*P* < 0.01, Fig. 3A and B), whereas FK866 administration significantly decreased the percentage of AQP4‐positive cells (*P* < 0.01, Fig. 3A and B).

### FK866 administration reduced the protein expression of inflammatory cytokines

**Figure 4:**
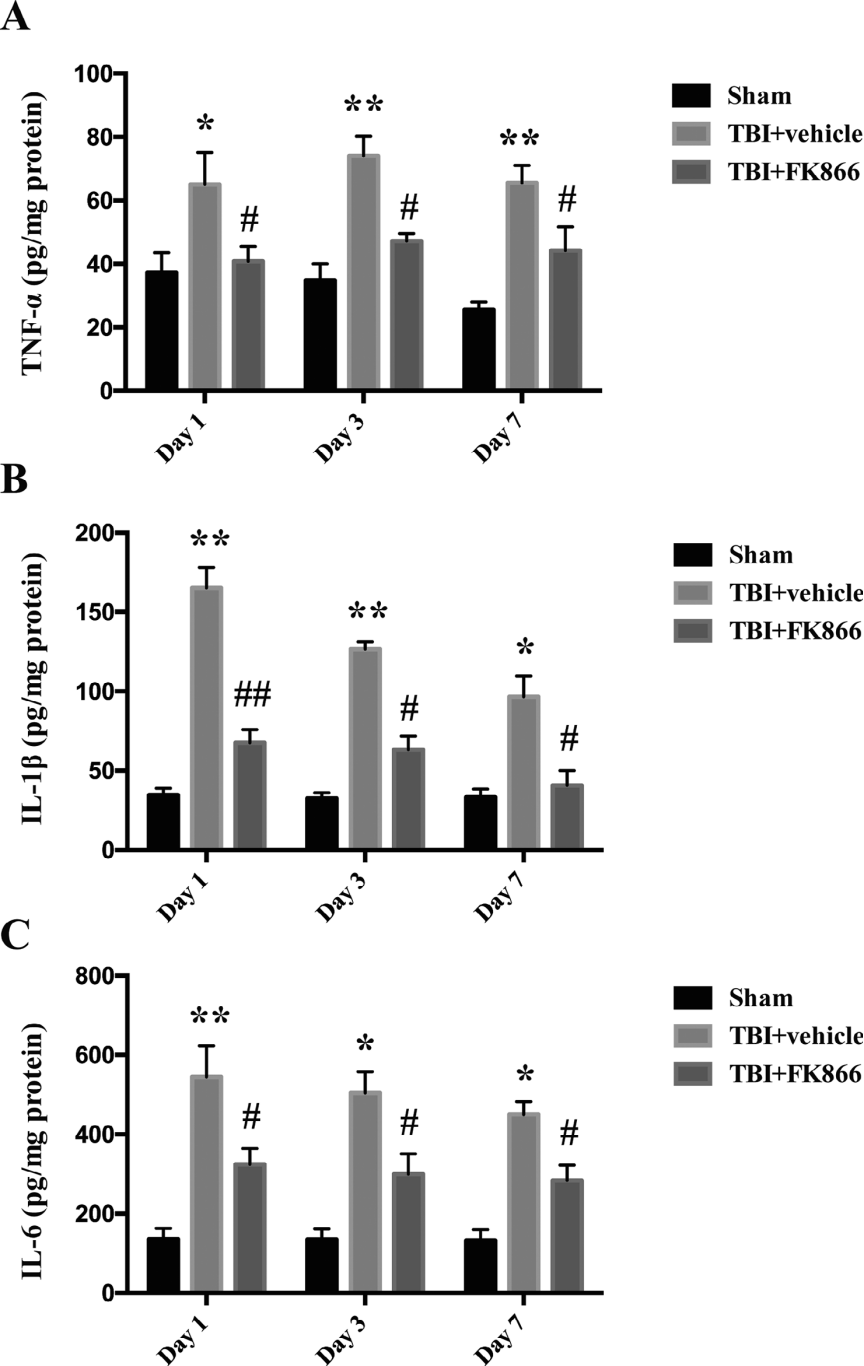
The effect of FK866 on the protein levels of proinflammatory cytokines in the peri‐injured cerebral cortex at 1, 3, and 7 days after TBI or sham operation. (A) The quantification of TNF‐α protein levels. The bar represents mean ± SD, *P < 0.05 versus sham, **P < 0.01 versus sham, **^#^** *P* < 0.05 versus TBI + vehicle. *n* = 6/group/time point. (B) The quantification of IL‐1β protein levels. The bar represents mean ± SD, *P < 0.05 versus sham, **P < 0.01 versus sham, **^#^** *P* < 0.05 versus TBI + vehicle, **^##^** *P* < 0.01 versus TBI + vehicle. *n* = 6/group/time point. (C) The quantification of IL‐6 protein levels. The bar represents mean ± SD, *P < 0.05 versus sham, **P < 0.01 versus sham. **^#^** *P* < 0.05 versus TBI + vehicle. *n* = 6/group/time point.

Next, we examined the effect of FK866 on proinflammatory cytokines, including TNF‐α, IL‐1β, and IL‐6, in the pericore cortex at day 1, day 3, and day 7 after TBI using ELISA kits. The inflammatory cytokines in sham rats at each time point after the operation were presented in a low background. The level of TNF‐α, IL‐1β, and IL‐6 exhibited significant increases at day 1 (*P* < 0.05 for TNF‐α, *P* < 0.01 for IL‐1β and IL‐6, Fig. 4), day3 (*P* < 0.01 for TNF‐α and IL‐1β, and *P* < 0.05 for IL‐6, Fig. 4), and day 7 (*P* < 0.01 for TNF‐α, *P* < 0.05 for IL‐1β and IL‐6, Fig. 4). FK866 administration significantly reduced the level of TNF‐α, IL‐1β, and IL‐6 at day 1 (*P* < 0.05 for TNF‐α and IL‐6, *P* < 0.01 for IL‐1β, Fig. 4), day3 (*P* < 0.05 for TNF‐α, IL‐1β, and IL‐6, Fig 4), and day 7 (*P* < 0.05 for TNF‐α, IL‐1β, and IL‐6, Fig. 4).

### FK866 decreased the number of microglia/macrophages and astrocytes

**Figure 5:**
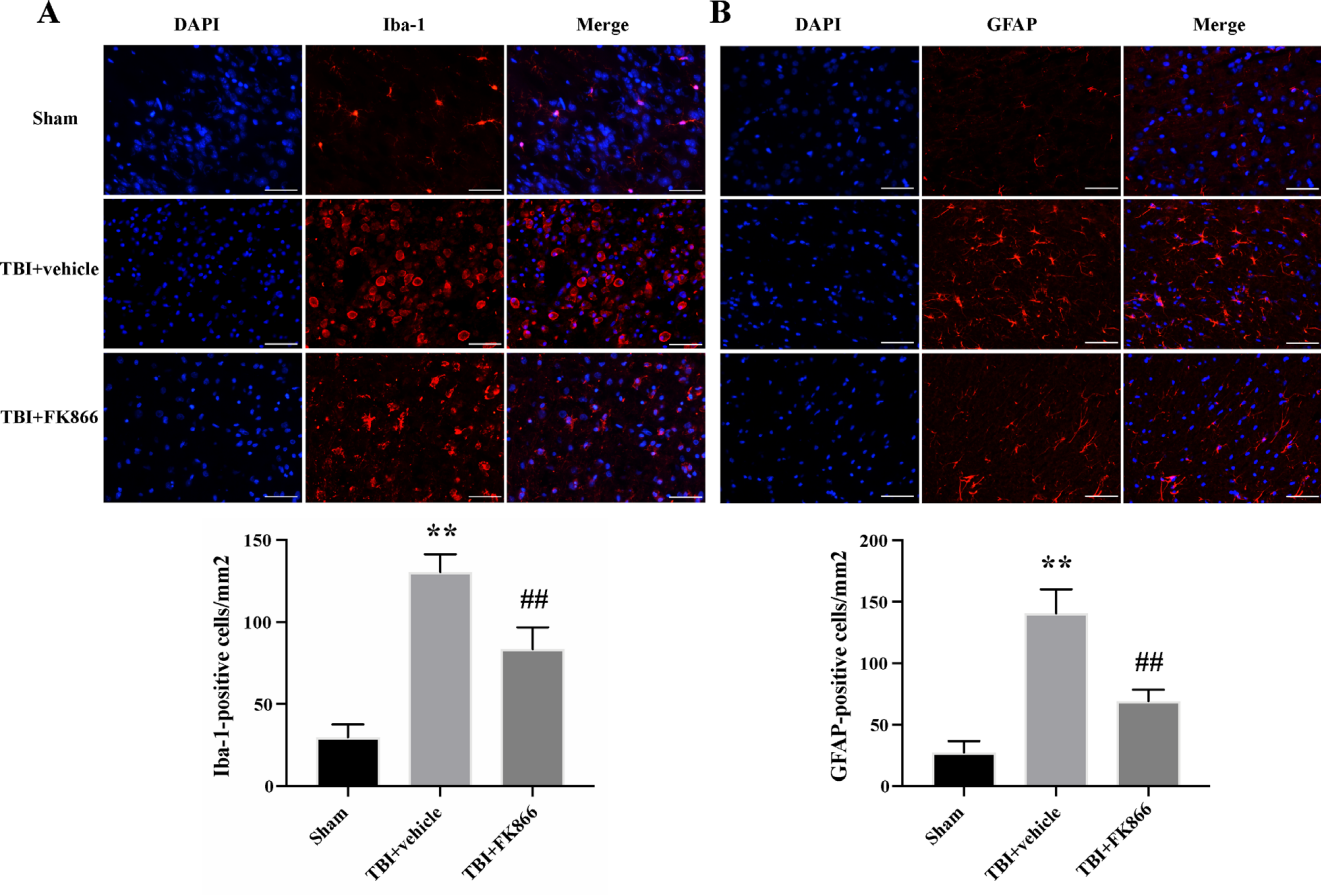
The effects of FK866 on the microgliosis and astrogliosis at 1 day after TBI. (A) The quantification of Iba‐1positive cells. In the rats subjected to TBI, the number of Iba‐1‐positive cells was higher when compared with the sham group, while FK866 decreased the number of Iba‐1‐positive cells. The bar represents mean ± SD. Scar bar = 100 μm. *n* = 5/group. **P < 0.01 versus sham group; ^##^ *P* < 0.01 versus TBI + vehicle group (B). The quantification of GFAP‐positive cells. In the rats subjected to TBI, the number of GFAP‐positive cells was higher when compared with the sham group, while FK866 decreased the number of GFAP‐positive cells. The bar represents mean ± SD. Scar bar = 100 μm. *n* = 5/group. **P < 0.01 versus sham group; ^##^P < 0.01 versus TBI + vehicle group.

The number of Iba‐1‐positive microglia/macrophages and GFAP‐positive astrocytes was significantly higher in the TBI + vehicle group than the control group (*P* < 0.01, Fig. 5), whereas FK866 administration significantly decreased the number of those cells (*P* < 0.01, Fig. 5).

### FK866 attenuated the nuclear translocation of NF‐κB p65

**Figure 6:**
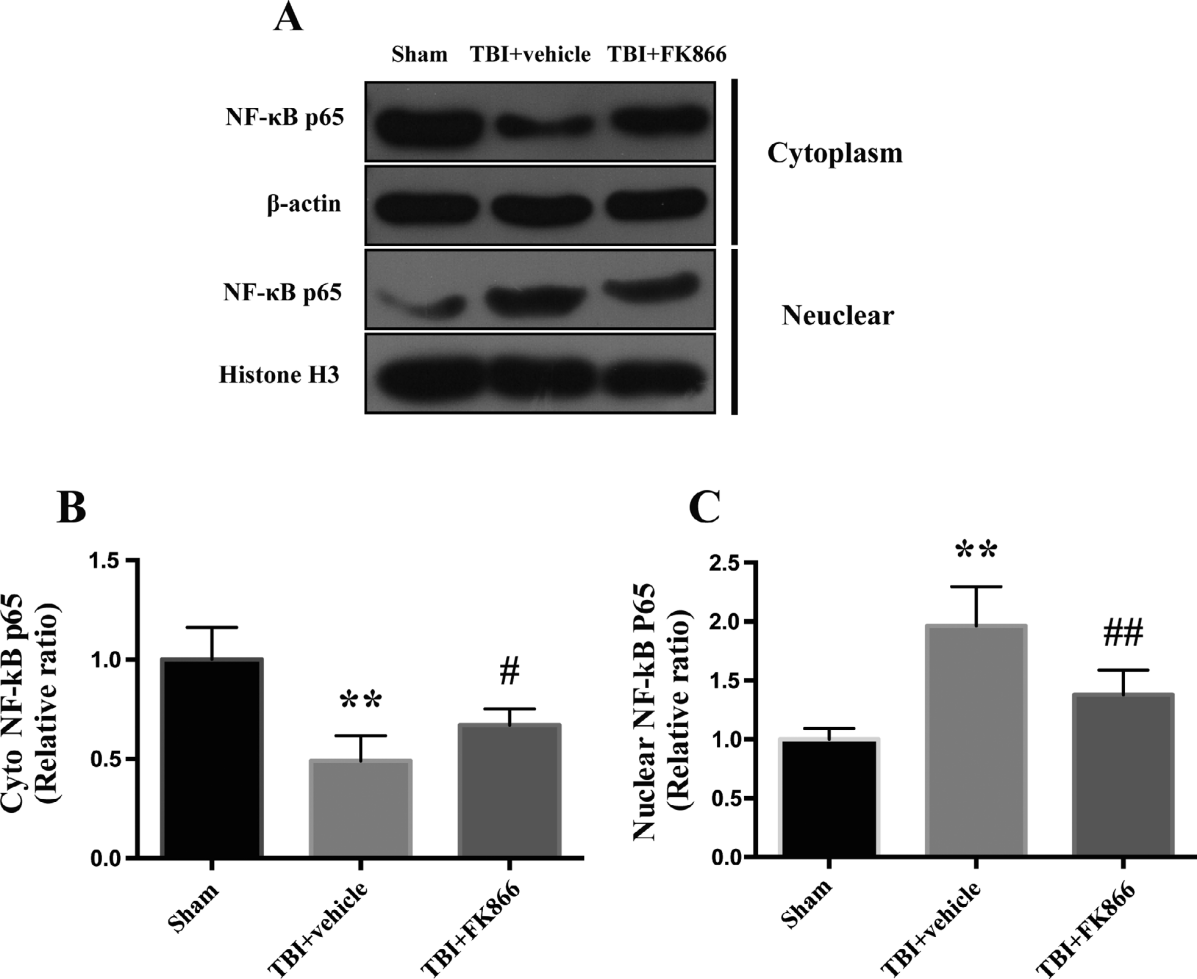
The effect of FK866 on nuclear translocation of NF‐κB p65 in the peri‐injured cerebral cortex at 24 h after TBI. (A) Representative western blots showing the levels of cytoplasmic and nuclear NF‐κB p65. (B) The densitometric quantification of cytoplasmic NF‐κB p65. *N* = 6. The bar represents mean ± SD, **P < 0.01 versus sham group; ^#^ *P* < 0.05 versus TBI + vehicle group. (C) The densitometric quantification of nuclear NF‐κB p65. *N* = 6. The bar represents mean ± SD, **P < 0.01 versus sham group; ^##^ *P* < 0.01 versus TBI + vehicle group.

Compared with the sham group, the cytoplasmic expression and the nuclear expression of NF‐κB p65 display opposite alteration: the former decreased significantly (*P* < 0.01, Fig. 6A and B) while the latter increased significantly (*P* < 0.01, Fig 6A and C) at 24 h post‐TBI. However, the administration of FK866 significantly upregulated its cytoplasmic expression (*P* < 0.05, Fig 6A and B) and decreased its nuclear expression (*P* < 0.01, Fig 6A and C).

### FK866 decreased neuronal apoptosis

**Figure 7:**
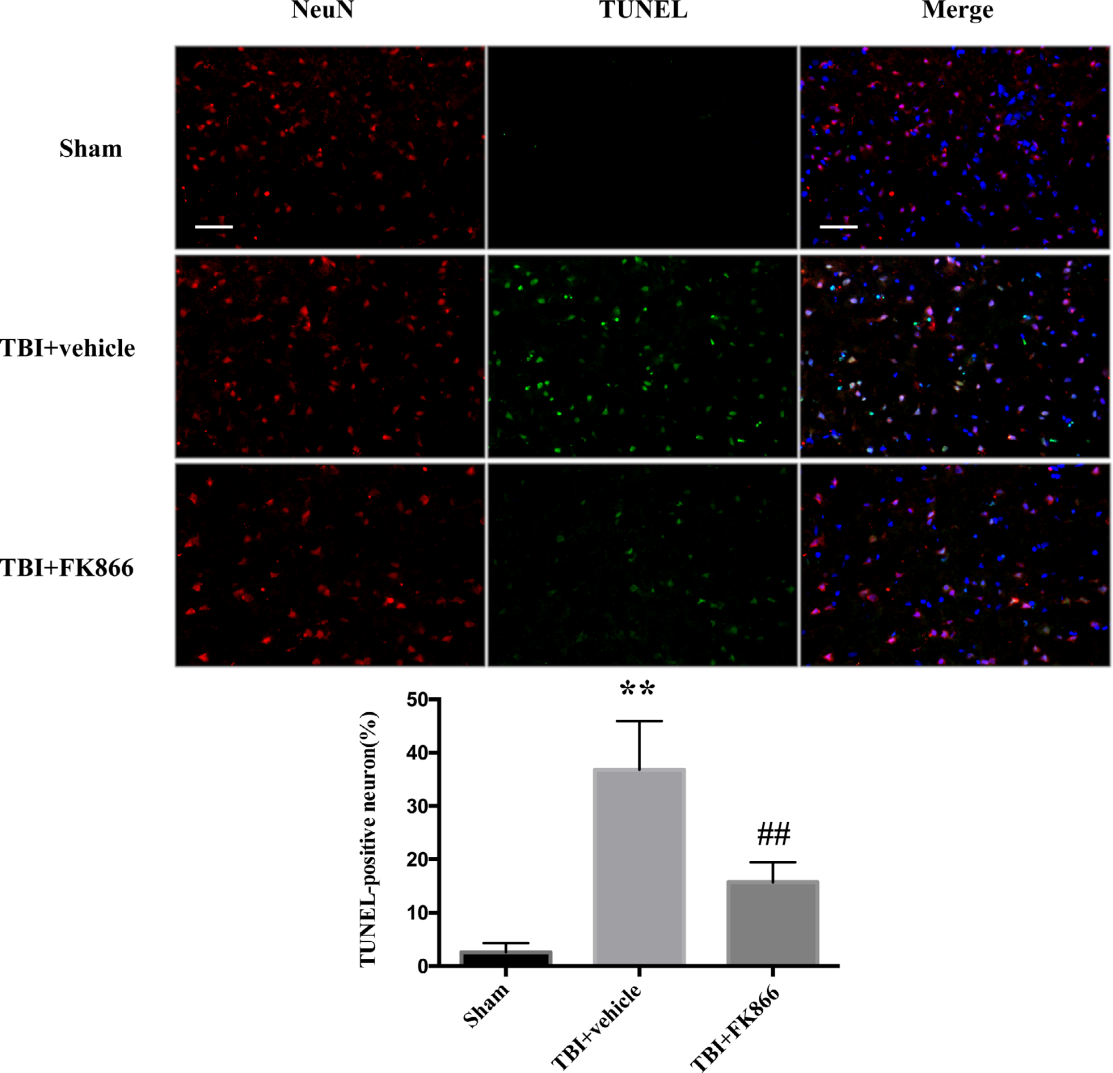
The effect of FK866 on neuronal neurons in the peri‐injured cerebral cortex at 24 h after TBI. In the rats subjected to TBI, the percentage of TUNEL‐positive neurons was higher when compared with the sham group, while FK866 decreased neuronal apoptosis. Scar bar = 50 μm. *n* = 5/group. The bar represents mean ± SD, **P < 0.01 versus sham group; ^##^P < 0.01 versus TBI + vehicle group.

The percentage of TUNEL‐positive neurons was significantly higher in the TBI + vehicle group than the control group (*P* < 0.01, Fig. 7A and B), whereas FK866 administration significantly decreased the percentage of TUNEL‐positive neurons (*P* < 0.01, Fig. 7A and B).

### FK866 enhanced the expression of Bcl‐2 and diminished the expression of Bax and caspase 3

**Figure 8:**
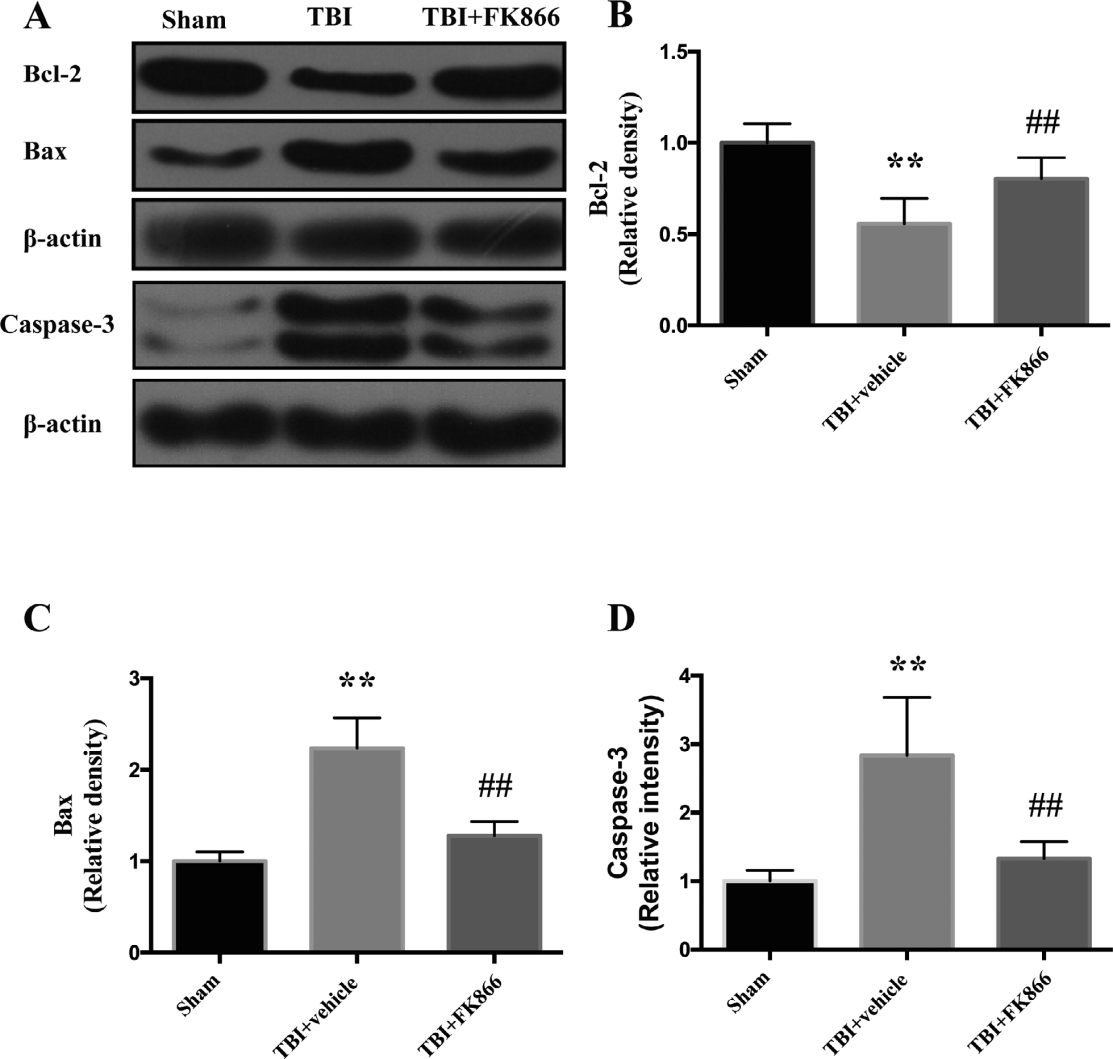
The effect of FK866 on expression of Bcl‐2, bax, and caspase 3 in the peri‐injured cerebral cortex at 24 h after TBI. (A) Representative western blots showing the levels of Bcl‐2, bax, and caspase 3. (B)The densitometric quantification of Bcl‐2. *n* = 6. The bar represents mean ± SD. **P < 0.01 versus sham group; ^##^ *P* < 0.01 versus TBI + vehicle group. (C) The densitometric quantification of Bax. *n* = 6. The bar represents mean ± SD. **P < 0.01 versus sham group; ^##^ *P* < 0.01 versus TBI + vehicle group. (D) The densitometric quantification of caspase 3. *n* = 6. The bar represents mean ± SD. **P < 0.01 versus sham group; ^##^ *P* < 0.01 versus TBI + vehicle group.

The content of Bcl‐2, Bax, and caspase 3 was examined to further confirm the antiapoptotic effects of FK866. By contrast with the sham group, the expression of Bcl‐2 was significantly reduced at 24h post‐TBI (*P* < 0.01, Fig. 8A and B), while FK866 administration upregulated its expression (*P* < 0.01, Fig. 8A and B). On the other hand, the expression of Bax and caspase 3 exhibited a significant increment in comparison with the sham group (*P* < 0.01, Fig. 8A, C and D), while the administration of FK866 significantly decreased their expressions (*P* < 0.01, Fig. 8A, C and D).

### FK866 inhibited p38 MAPK and ERK activation

**Figure 9:**
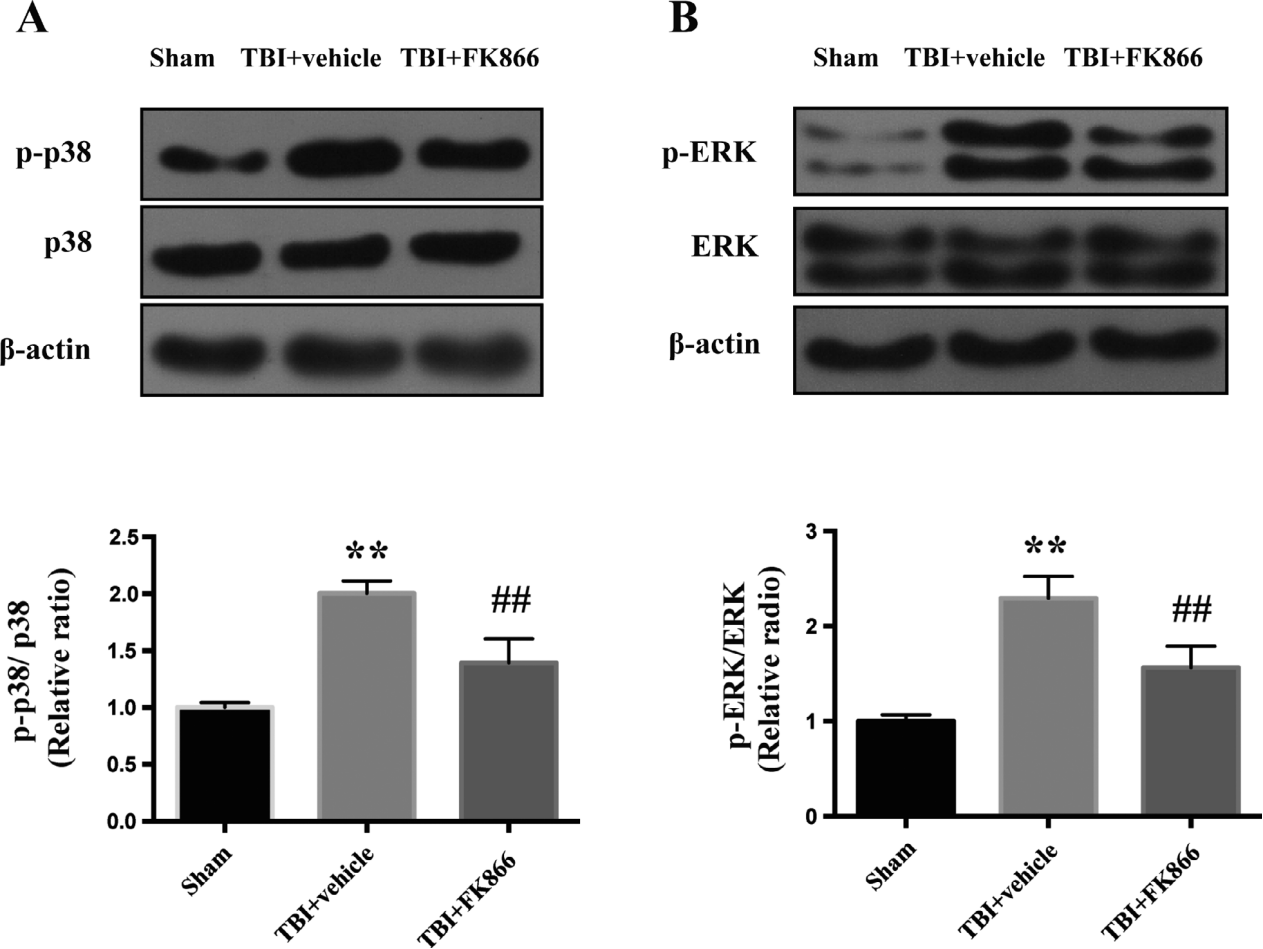
The effect of FK866 on p38/ERK MAPKs activation in the peri‐injured cerebral cortex at 24 h after TBI. (A) Representative western blots showing the p38/ERK MAPKs activation. (B) The densitometric quantification of p‐p38/p38 ratio. *N* = 6. The bar represents mean ± SD, **P < 0.01 versus sham group; ^##^ *P* < 0.01 versus TBI + vehicle group. (C) The densitometric quantification of p‐ERK/ERK ratio. *N* = 6. The bar represents mean ± SD. **P < 0.01 versus sham group; ^##^ *P* < 0.01 versus TBI + vehicle group.

To explore the underlying mechanism, western blot was utilized to examine phosphorylated p38 MAPK and ERK at the protein level. Compared with the sham group, the expression of phosphorylated p38 MAPK and ERK increased significantly at 24 h post‐TBI (*P* < 0.01, Fig. 9A and B), while administration of FK866 significantly decreased their expressions (*P* < 0.01, Fig. 9A and B).

## Discussion

**Figure 10:**
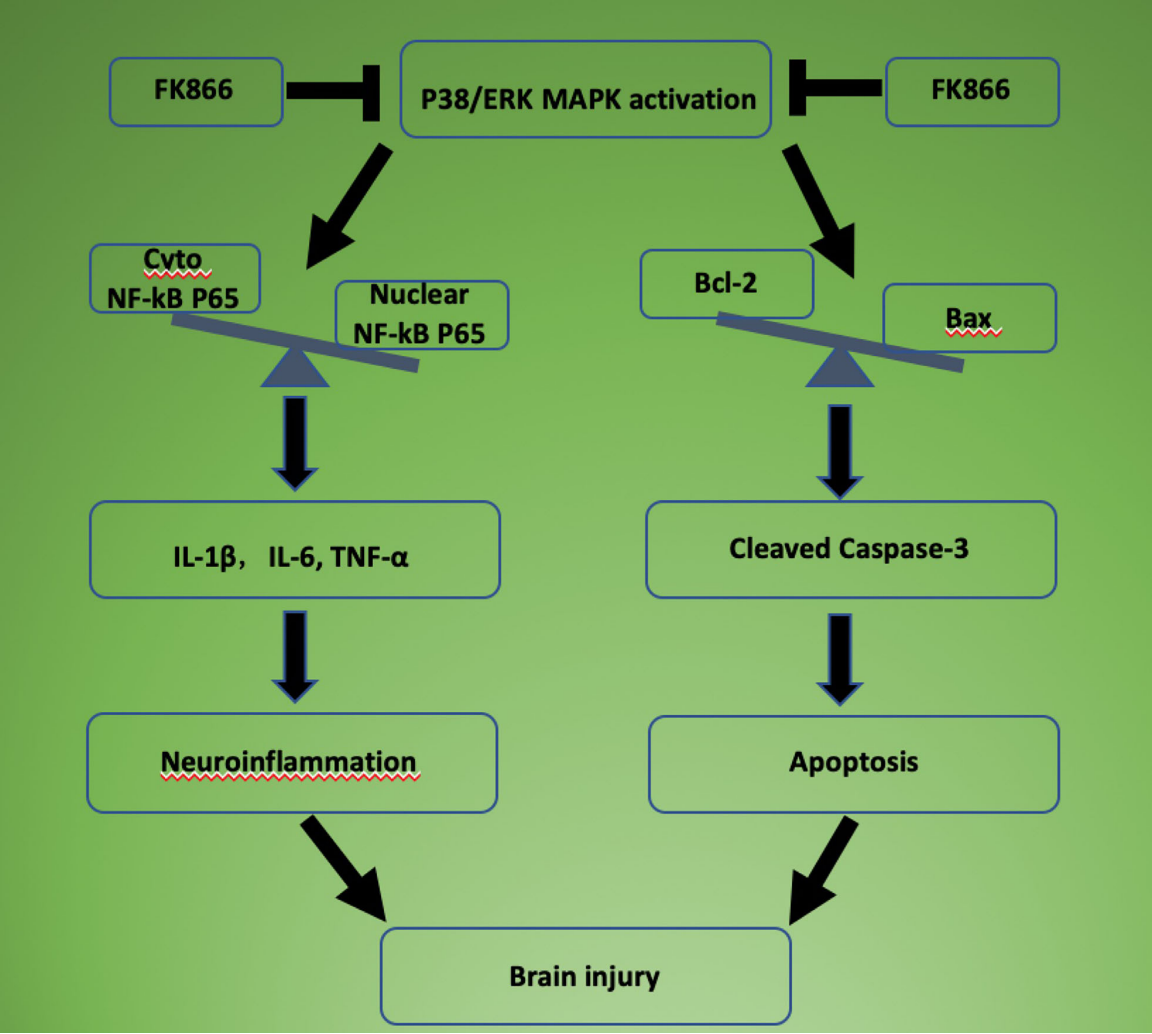
Schematic diagram shows potential molecular mechanisms of FK866‐induced neuroprotection against TBI.

In this study, several novel founding presented as follow: (a) FK866 prevented brain edema, BBB disruption, and improved neurological function after TBI. (b) FK866 reduced the number of microglia/macrophages and astrocyte, downregulated the level of proinflammatory cytokines and proapoptotic proteins, and upregulated the levels of antiapoptotic protein. (c) FK866 attenuated TBI‐induced neuroinflammation and apoptosis, and the potential mechanisms, at least in part, involved p38/ERK MAPKs signaling pathway (Fig. 10).

TBI refers to the impairment of brain function resulting from an external force such as impact or penetration.^20^ Brain edema following TBI is an important factor which contributes to the evolution of brain injury and is associated with significant morbidity and mortality.^21^, ^22^ It is classified as vasogenic or cytotoxic edema according to BBB disruption or dysfunction of cellular ionic pumps. Both vasogenic edema and cytotoxic edema were found in TBI patients by MRI.^23^ AQP4 plays an important role in maintaining brain water homeostasis, mediating the flux of water at the interface between the cerebral vasculature.^24^ AQP4 is known to predominantly contribute to cytotoxic edema after TBI.^25^ In our study, we found that FK866 not only prevented the BBB disruption but also downregulated the expression of AQP4. In addition, neuroinflammation is closely connected with BBB disruption: BBB disruption fuels neuroinflammation, and on the other hand, neuroinflammation aggravate BBB disruption. Astrocytes and microglia/macrophages are often the primary cell types to initiate an inflammatory cascade upon sensing damage.^26^ In this study, we found that astrocytes and microglia/macrophages increased significantly after TBI, which were reduced by FK866 treatment. In addition, proinflammatory cytokines were also decreased by FK866 administration. NF‐κB is a classic transcription factor that exerts regulatory effects on proinflammatory cytokines such as TNF‐α, IL‐1β, and IL‐6 at the transcriptional and translational levels.^27^ Previous studies indicated that NF‐κB activation participated in the secondary brain injury post‐TBI.^28^, ^29^ In our study, we found NF‐κB activation after TBI, which was inhibited by FK866 administration. In addition, neuronal apoptosis increased, which was also decreased by FK866 treatment.

The mitogen‐activated protein kinases (MAPKs) pathway is an important factor regulating the release of proinflammatory cytokines and apoptosis.^30^ MAPK is a family of serine/threonine protein kinases, including ERK, JNK, and p38. Previous studies indicated that activation of NF‐κB was closely associated with MAPK signaling pathway.^31^, ^32^ The activation of MAPK led to the upregulation of caspase 3 and the decline in Bcl‐2, thus engendering apoptosis.^33^ It was observed that phosphorylated ERK and p38, rather than JNK, augmented rapidly after TBI in a cortical impact model of mice,^34^ while the findings of Chen et al. indicated that the activation of JNK and p38, instead of ERK, was enhanced in the cortical neurons after traumatic injury.^35^ Nevertheless, in a lateral fluid percussion model, phosphorylated ERK and JNK, other than p38, demonstrated an increase in the cortex and hippocampus of the TBI rats.^36^ Our results showed that the phosphorylation of ERK and p38 was intensified at 24 h after TBI. And, we also found that FK866 reduced phosphorylated levels of p38 and ERK. Taken together, these observations suggest that FK866 attenuated TBI‐induced neuroinflammation and apoptosis, at least in part, through p38/ERK MAPKs signaling pathway.

There did exist some limitations in this study. First, Fk866 exhibited neuroprotective effects in female TBI rats, but the potential mechanisms are still needed. Second, we examined the effects and possible mechanisms of FK866 in a focal TBI model, studies in global events such as those mimicked by fluid percussion brain injury are still required.

This study demonstrated that NAMPT inhibitor FK866 attenuated neuroinflammation, apoptosis, BBB disruption, and brain edema, and improved neurological function following TBI. The p38/ERK MAPK signaling pathway may be involved in the anti‐inflammatory and antiapoptotic effects of FK866 following TBI.

## Conflict of Interest

The authors declare that they have no conflict of interest.

## Supporting information


**Figure S1.** The typical image of traumatic brain injury model.

**Figure S2.** The experimental outline in this study.

**Figure S3.** The number of rats sacrificed in this study.

**Figure S4.** The effect of FK866 on neurological function after TBI in female rats.
