# Supplementary figures and images for: Neuroprotective effects of FK866 against traumatic brain injury: Involvement of p38/ERK pathway
Source: Ann Clin Transl Neurol. 2020 Apr 17;7(5):742–56. doi: 10.1002/acn3.51044 (PMC7261767; doi:10.1002/acn3.51044)

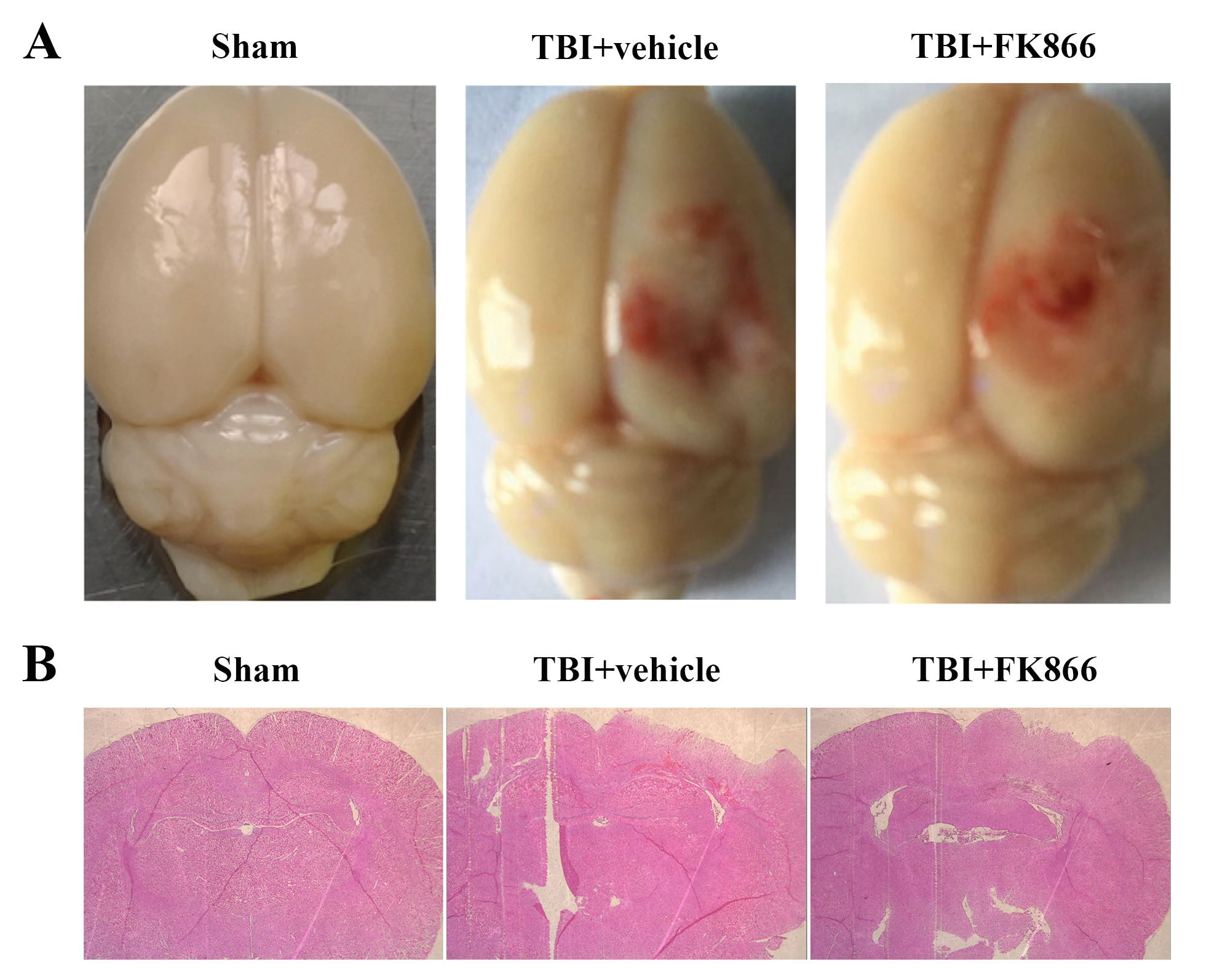

Supplement: Supplementary file 1 — Figure S1. The typical image of traumatic brain injury model. [file ACN3-7-742-s001.tif]

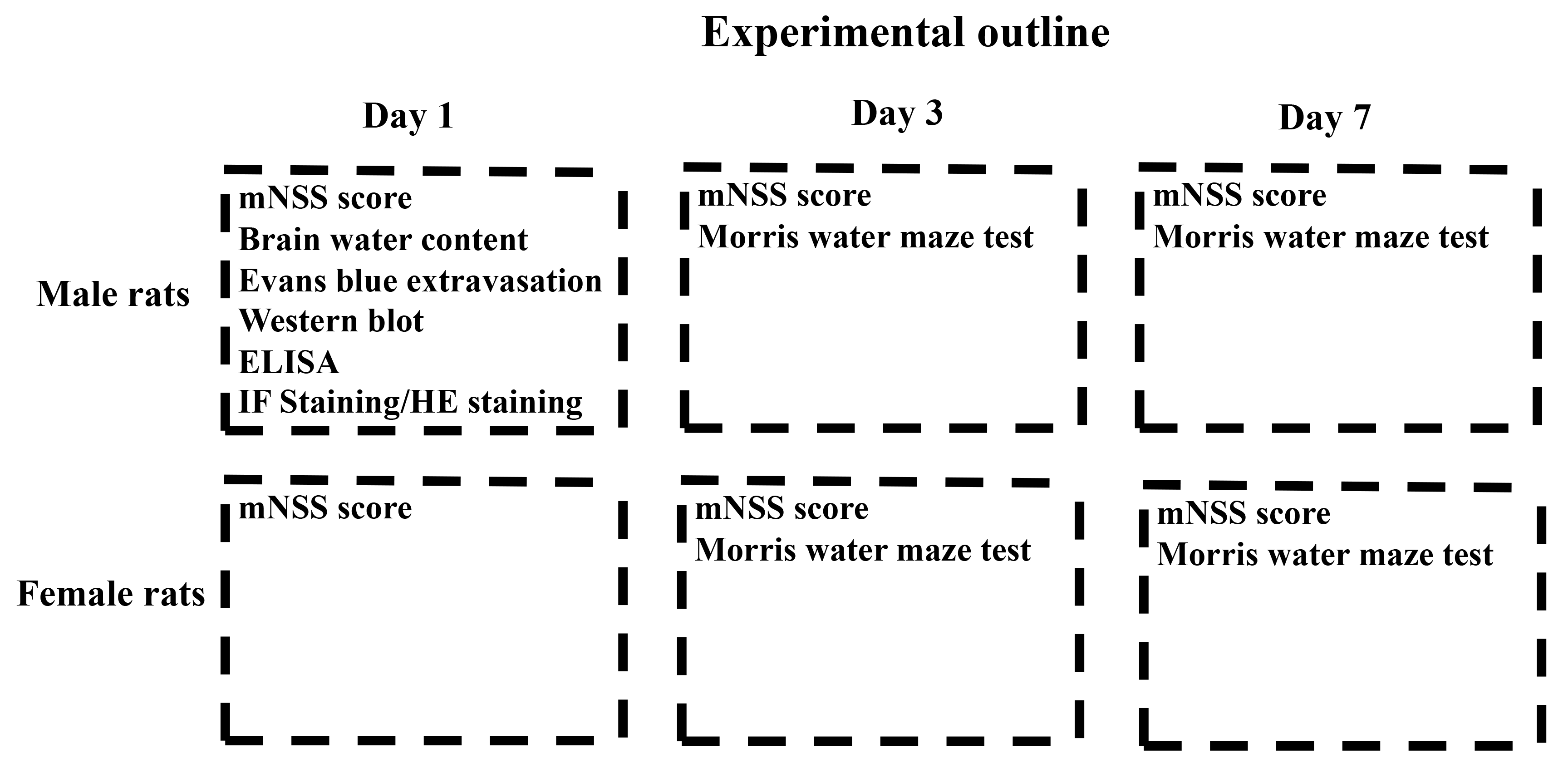

Supplement: Supplementary file 2 — Figure S2. The experimental outline in this study. [file ACN3-7-742-s002.tif]

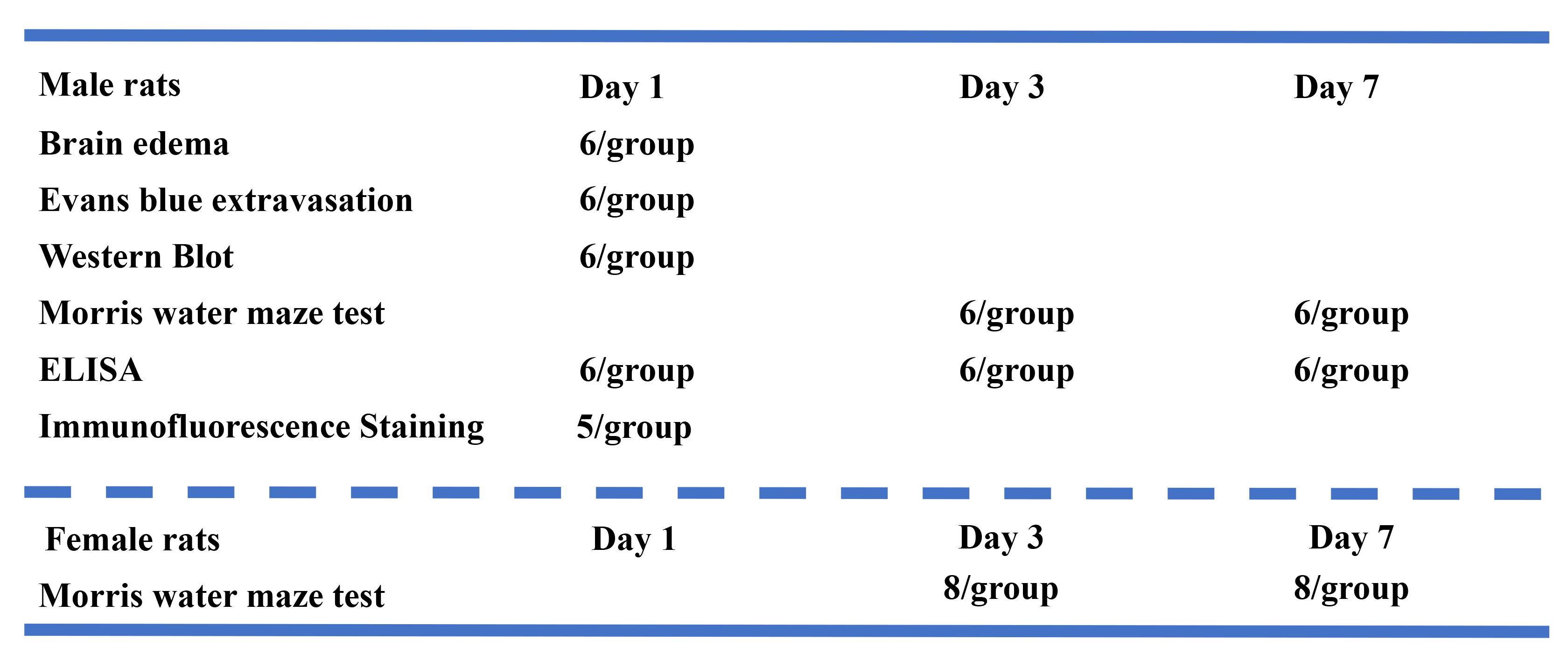

Supplement: Supplementary file 3 — Figure S3. The number of rats sacrificed in this study. [file ACN3-7-742-s003.tif]

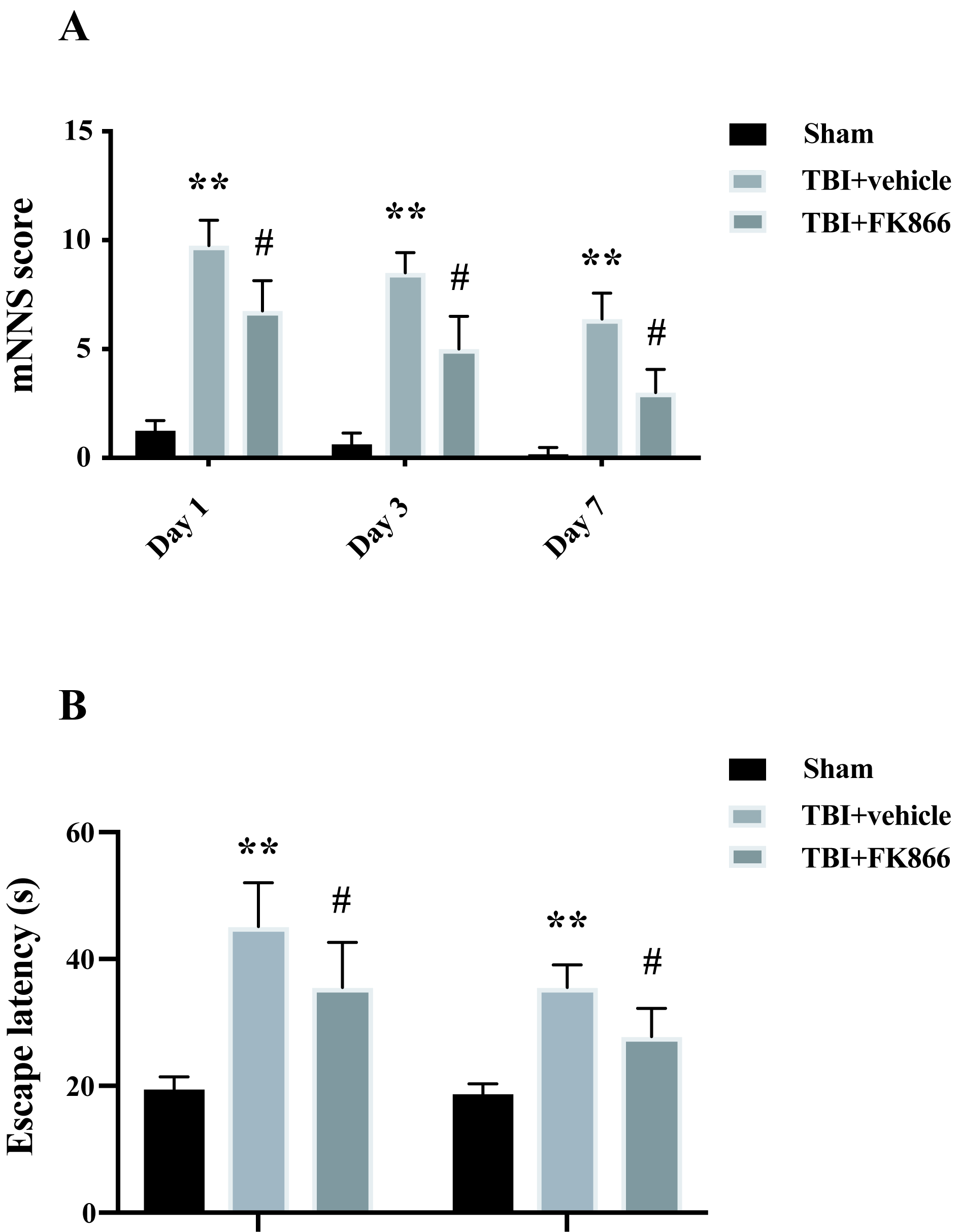

Supplement: Supplementary file 4 — Figure S4. The effect of FK866 on neurological function after TBI in female rats. [file ACN3-7-742-s004.tif]
